# Combining RNAscope, Immunohistochemistry (IHC) and Digital Image Analysis to Assess Podoplanin (PDPN) Protein and PDPN_mRNA Expression on Formalin-Fixed Paraffin-Embedded Normal Human Placenta Tissues

**DOI:** 10.3390/cimb46060310

**Published:** 2024-05-24

**Authors:** Larisa Cristina Tomescu, Andrei Alexandru Cosma, Mihaela Pasca Fenesan, Eugen Melnic, Vergil Petrovici, Simona Sarb, Monica Chis, Ioan Sas, Domenico Ribatti, Anca Maria Cimpean, Florica Ramona Dorobantu

**Affiliations:** 1 Doctoral School in Medicine, Victor Babes University of Medicine and Pharmacy, 300041 Timisoara, Romania; tomescu.larisa@umft.ro (L.C.T.); cosma.andrei@umft.ro (A.A.C.); fenesan.mihaela@gmail.com (M.P.F.); 2 Department of Obstetrics and Gynecology, Victor Babes University of Medicine and Pharmacy, 300041 Timisoara, Romania; sasioan56@yahoo.com; 3 Department of Microscopic Morphology/Histology, Victor Babes University of Medicine and Pharmacy, 300041 Timisoara, Romania; sarb.simona@umft.ro; 4 OncoHelp Hospital, 300239 Timisoara, Romania; 5 Department of Pathology, Nicolae Testemitanu State University of Medicine and Pharmacy, 2004 Chisinau, Moldova; eugen.melnic@usmf.md (E.M.); petrovicivergil64@gmail.com (V.P.); 6 Department ME2/Rheumatology, Rehabilitation, Physical Medicine and Balneology, Faculty of Medicine, George Emil Palade University of Medicine, Pharmacy, Science, and Technology of TârguMureş (UMPhST), 540088 Targu Mures, Romania; monicacopotoiu@gmail.com; 7 Clinic of Rheumatology, Emergency County Hospital of Târgu Mureş, 540088 Targu Mures, Romania; 8 Department of Translational Biomedicine and Neuroscience, University of Bari Medical School, 70124 Bari, Italy; domenico.ribatti@uniba.it; 9 Center of Expertise for Rare Vascular Disease in Children, Emergency Hospital for Children Louis Turcanu, 300011 Timisoara, Romania; 10 Department of Neonatology, Faculty of Medicine and Pharmacy, University of Oradea, 410087 Oradea, Romania; rdorobantu@uoradea.ro

**Keywords:** podolanin, placental villi, decidua, normal human placenta

## Abstract

The expression and function of podoplanin (PDPN) in the normal human placenta has been debated in placental evaluation. This study emphasizes the importance of a multimodal approach of PDPN expression in normal human placentas. A complete examination is performed using immunohistochemistry, RNAscope and automated Digital Image examination (DIA) interpretation. QuPath DIA-based analysis automatically generated the stromal and histological scores of PDPN expression for immunohistochemistry and RNAscope stains. The umbilical cord’s isolated fibroblasts and luminal structures expressed PDPN protein and PDPN_mRNA. RNAscope detected PDPN_mRNA upregulation in syncytial placental knots trophoblastic cells, but immunohistochemistry did not certify this at the protein level. The study found a significant correlation between the IHC and RNAscope H-Score (*p* = 0.033) and Allred Score (*p* = 0.05). A successful multimodal strategy for PDPN assessment in human placentas confirmed PDPN expression heterogeneity in the full-term human normal placenta and umbilical cord at the protein and mRNA level. In placental syncytial knots trophoblastic cells, PDPN showed mRNA overexpression, suggesting a potential role in placenta maturation.

## 1. Introduction

Podoplanin (PDPN) is a transmembrane protein of the mucin type, with a molecular weight ranging from 36 to 43 kilodaltons. The gene has homologous counterparts in humans, mice, rats, dogs and hamsters, and it exhibits a considerable degree of conservation across different species. PDPN plays a diverse range of roles, such as controlling the growth of organs, facilitating cell movement and promoting the formation and spread of tumors [1,2,3,4]. A variety of cell types, including lymphatic endothelial cells [5,6], fibroblasts [7], mesothelial cells [8] and basal cells of stratified epithelia [9], can be identified at the protein and gene level by using several methods such as immunohistochemistry [10] or molecular techniques [11,12,13].

Most of the studies regarding PDPN were performed on human adult normal and pathologic bioptic specimens. PDPN involvement in the embryonic development and prenatal stages is rarely known for human tissues but extensively characterized on various animal experimental models, such as in mice [14], rabbits [15] or even animals that are not used for experimental purposes, such as sea lions [16], Tasmanian devils [17] or bears [18]. This high interest in developing PDPN antibodies for usual or rare species supports the importance of PDPN in the development and proper function of several normal tissues.

On an advanced search on PubMed regarding PDPN expression in placenta, only 19 papers were listed [19], but only 6 out of the 19 were focused on normal human full-term placenta [20], with a special emphasis on lymphatic vessels, whose existence in human placenta is highly controversial. No direct evidence regarding PDPN expression and distribution into the human umbilical cord has been yet reported.

Recently, it has been suggested that there is an expression of podoplanin in the cells of the umbilical cord [21], the fetus [22] and the maternal placenta [23]. Literature data concerning podoplanin expression in human placentas and umbilical cords are controversial, as well as that concerning the presence of lymphatic vessels in the normal human placenta [23,24].

The interest in the PDPN expression in human placenta continuously increased based on several papers which reported its importance in the preeclampsia mechanism.

Mice models of preeclampsia have shown that lymphatic channels are present in placentas, being involved in the lymphocyte trafficking across the endothelium [25]. Moreover, different gene expression patterns for lymphatic endothelial cells have been demonstrated in between placental tissue from patients with preeclampsia and normal placenta [26]. Although podoplanin expression in umbilical cord mesenchymal cells has not been confirmed, reports of its existence in mesenchymal stem cells from adipose tissue [27] and other sources have been linked to lymphangiogenesis.

The most frequently used technique for assessing PDPN expression in human placenta is immunohistochemistry (IHC) [22,23,24], followed by Western Blot [22] and, to a lesser extent, gene expression techniques [26]. According to The Human Protein Atlas, PDPN protein expression (detected by IHC) is restricted to the stromal cells from placental villi but is lacking in trophoblast cells [28]. Also, The Human Protein Atlas PDPN_RNA expression in human normal placenta is not reported [28]. Conventional methods for detecting and quantifying mRNA expression, such as the Polymerase Chain Reaction (PCR) technique, need the isolation of individual cells from their natural environment [29]. However, these techniques can “hide” the variations in gene expression between cells and may lead to the omission of crucial information, particularly regarding the spatial arrangement of the cells being analyzed [30]. In situ hybridization techniques were applied on human placenta tissue [31] but not for the detection and characterization of PDPN expression/distribution in the stromal and epithelial components of the human placenta. Among these methods, RNAscope is a commercially accessible in situ hybridization (ISH) technique used to identify RNA in formalin-fixed paraffin-embedded tissue [13,32,33,34]. This technique enables the visualization of single RNA molecules within individual cells. Its efficacy has been examined and confirmed in several human medical conditions [13,32,33,34]. Crucially, the main distinction from the usual RNA ISH is that RNAScope identifies target-specific probes, reducing the occurrence of non-specific off-target signals. As a result, it produces extremely specific staining [35,36].

The subjective visual assessment of the density and intensity of the immunohistochemical staining (typically indicated as +1/low, +2/moderate or +3/high) or the number of positive dots for the RNAscope, which are manually counted by researchers and interpreted in accordance with the manufacturer’s protocol, forms the basis of empirical and non-standardized “scores” for the interpretation of immunohistochemical and RNA ISH results. All these issues may influence the impact of the results, but they may be counterbalanced using digital image analysis programs which perform the interpretation of IHC and RNA scope results by the automated counting of positive signals combined with the delivery of more accurate results.

Neither RNAscope nor digital image analysis methods were used for PDPN quantification in human full-term placenta. The aim of the present study is to visualize and characterize PDPN expression in the human normal placenta by combining IHC, RNAscope and Digital Image Analysis (DIA) for the accuracy improvement of PDPN in the normal full-term human placenta.

## 2. Materials and Methods

### 2.1. Patients, Samples and Inclusion and Exclusion Criteria

This is a retrospective study including, initially, 100 human placenta and adjacent umbilical cord tissue specimens collected from patients aged between 23 and 35 years old in between 2021 and 2023. A total of 30 out of 100 human placentas were eligible for this study. The inclusion criteria for this selection were: (1) normal fetal and placental development based on clinical, biological and ultrasound parameters periodically evaluated during pregnancy; (2) no history of preeclampsia or other pathologies with an impact on placenta development; (3) no hemorrhagic events during pregnancy; (4) no placenta implantation pathology; (5) no labor-associated events; (6) natural delivery of the placenta; (7) the normal macroscopy of the placenta after delivery; (8) live fetuses with Apgar scores of 9 and 10; (9) normal histology of the placenta and umbilical cord during microscopic assessment; (10) positive immunohistochemical staining for vimentin as a marker of proper tissue fixation—mandatory for the following IHC and RNAscope techniques. The exclusion criteria were focused on any pathology reported during pregnancy but mostly on the selection of tissues for IHC and RNAscope: (1) incorrect tissue fixation related to the formalin type and fixation time; (2) damaged tissues during primary processing steps; (3) the detection of microscopic pathologies of the human placenta for the initial evaluation of hematoxylin- and eosin-stained specimens; (4) lack of positivity for vimentin detected tissue specimens improper for RNAscope; (5) placenta from a C-section.

Informed consent from each patient, in accordance with the criteria of the Helsinki Declaration on the conduct of research on human tissues, had been obtained.

The present study obtained the approval of the Ethics Committee of “Victor Babes” University of Medicine and Pharmacy in Timisoara with No. 69/2020.

### 2.2. Primary Processing and Histopathological Analysis

The obtained placental tissues and umbilical cord fragments have been fixed in 10% buffered formalin for a period of 24/48 h. Fixed tissue samples were later included in the paraffin following the standard protocol for this technique. Three micrometer serial sections were obtained for each case, and the routine hematoxylin and eosin stain was performed to reevaluate the microscopic criteria for the selection of the normal human placenta and umbilical cord. The microscopic inclusion criteria for the specimens’ selection were: (1) the presence of chorionic villi with a stromal and trophoblast compartment; (2) the presence of decidual cells; (3) the absence of inflammation in the stromal compartment; (4) the absence of other cellular changes induced by preeclampsia or specific for any viral and/or bacterial infections; (5) normal microscopy of the umbilical cord sections with the presence of cellular and extracellular matrix components of the mucous connective tissue and the presence of the umbilical vessels; (6) the absence of any cellular and nuclear changes given by the improper fixation and/or deficiency in the primary processing of the tissue biopsies. Two experienced pathologists independently revised the slides, and the final decision about the case selection was given by both after discussing their preliminary results and reaching a final common agreement.

### 2.3. PDPN Immunohistochemistry

In the pre-analytical step of the immunohistochemistry technique, we performed vimentin (Clone V9, DakoCytomation, Santa Clara, CA, USA) staining for all specimens to check the quality of the tissue and to select the proper ones for immunohistochemical staining. The specimens that lack vimentin immunostaining were excluded from the study due to the improper quality of the tissue for the IHC and RNAscope methods. A vimentin-positive reaction in the mesenchymal cells of the placental chorion and umbilical cord was considered as a positive tissue criterion that was valid for the subsequent application of immunohistochemical podoplanin staining.

Immunostaining for PDPN (Clone D2-40, redy-to-use mouse monoclonal antibody, DakoCytomation, CA, USA) was performed following the following consecutive steps: (1) endogenous peroxidase block by using 3% hydrogen peroxide for 5 min; (2) antigen unmasking by using pH 6 unmasking buffer solution for 30 min; (3) thirty minutes of incubation with a primary antibody (D2-40) at room temperature; (4) secondary step amplification by applying the Bond Max Refine Detection System (Leica Microscope Systems, Cambridge, UK); (5) visualization by using 3, 3′ diaminobenzidine as the chromogen; (6) nuclear counterstain with hematoxylin; (7) mounting of the slides with permanent mounting media. All immunohistochemical procedures starting from dewaxing to the mounting step were fully automated using the Bond Max Autostainer (Leica Microscope Systems, Cambridge, UK) and related software capable of controlling every step of the immunohistochemical technique from dewaxing to hematoxylin staining. The brown positive signals with cytoplasmic localization in the placental villi stromal and trophoblast compartments and also in the endothelial and mesenchymal cells from both the placenta villi and umbilical cord were considered and quantified.

### 2.4. RNAscope Technique

Briefly, to prepare FFPE specimens for the RNAscope procedure, the tissues were fixed and permeable to give the hybridization sample access to the cellular epitope being studied. The two-hour hybridization phase performed at 40 °C was followed by multistep signal amplification performed by the sequential application of reagents, followed by the visualization of RNA as brown point signals visualized by the application of diaminobenzidine.

The detection sensitivity of the RNAscope method has been proven by using positive (POLR2A) and negative controls (probes against the bacterial *dap B* gene), evaluated using the same protocol applied in our previous studies [32,33,34,35].

The manufacturer provided an interpretation protocol of podoplanin_mRNA (PDPN_mRNA) amplification based on a semi-quantitative method. According to this protocol, PDPN_mRNA amplification was qualified in five degrees as 0 (no staining or fewer than 1 point at every 10 cells, 40× magnification), 1 (1–3 points/cell visible at 20–40× magnification), 2 (4–10 points/cell; very few groups of points visible at 20–40× magnification), 3 (>10 points/cell; less than 10% of positive cells have groups of points visible at 20× magnification) and 4 (>10 dots/cell; more than 10% of positive cells have groups of visible points at 20× magnification). This protocol is hard to apply and is highly subjective; thus, we chose to perform a Qu Path analysis of positive PDPN_mRNA signals, and we obtained the H-Score and Allred Score based on the intensity and density of positive signals. The H-Score and Allred Score for RNAscope were correlated with similar parameters derived from the QuPath analysis performed for IHC-stained specimens.

### 2.5. Assessement and Scoring of PDPN by Using Qu Path Analysis 

Microscopic quantification of the density and intensity of PDPN+ cells in the umbilical cord and placenta at term was performed using the Qu Path automated interpretation program, which allowed for the application of the intensity and density scores like the Allred score used in the quantification of hormone receptors in breast cancer. Based on this score, the Qu Path program allowed for the identification of PDPN+ cells as well as their classification based on the immunohistochemical expression intensity of D2-40.

IHC-stained slides were scanned using a Grundium OCUS 20 microscope (Grundium, Tampere, Finland) and stored in the Case Center Slide Library as svs. files (3DHistech, Budapest, Hungary). A project was created by importing all the D2-40-colored slides to Qu Path Image Analysis System version 0.4.3 [19], an open-source platform for microscopic slide bioimage analysis, where they were examined using integrated software and additional programs. In short, we selected three to five regions of interest (ROIs) from the tumor stroma using the instruments in the toolbar that gave us the most accurate delimitation of placental and umbilical cord areas that contained PDPN+ cells. The analysis then proceeded with the detection of the cell by choosing a positive cell detection option and setting cell parameters and intensity parameters. The detection image was set for a sum of optical density with a requested pixel size of 0.5 μm and a cell expansion of 1.988 μm, excluding the nucleus. The intensity threshold parameters comprised the overall score and the three threshold levels assessed as low (+1, highlighted in yellow), moderate (+2, highlighted in orange) and high (+3, highlighted in red), with all cells selected in blue considered negative. During the automatic score assessment, the Qu Path software version 0.4.3 offered us the number of cells, the density score and the intensity score separately and also a combined stromal score (SS, like the Allred score, combining the intensity and density of detected PDPN+ cells and PDPN_mRNA brown-stained dots). Also, an H score was included in the final assessment performed by Qu Path analysis, representing the histological score.

### 2.6. Microscopic Image Acquisition and Analysis and Statistical Data Analysis

The histological specimens stained morphologically, immunohistochemically and by the technique of in situ hybridization with chromogens were scanned using the OCUS scanning microscope and archived in the Slide Center digital slide library. The semiquantitative evaluation of the immunohistochemistry and in situ hybridization results was performed using Qu Path computational pathology software version 0.4.3 and the software semiquantitative evaluation systems of this program.

### 2.7. Statistical Analysis 

Statistical analysis was performed automatically by exporting the data from the Qu Path system to the Jamovi statistical analysis system. We applied statistical tests to check if protein expression and mRNA expression are significantly correlated. A *p*-value less than 0.05 was considered statistically significant, while a *p* value less than 0.001 was interpreted as strongly significant.

## 3. Results

### 3.1. Immunohistochemical and mRNA Expression of PDPN in the Umbilical Cord

**Figure 1:**
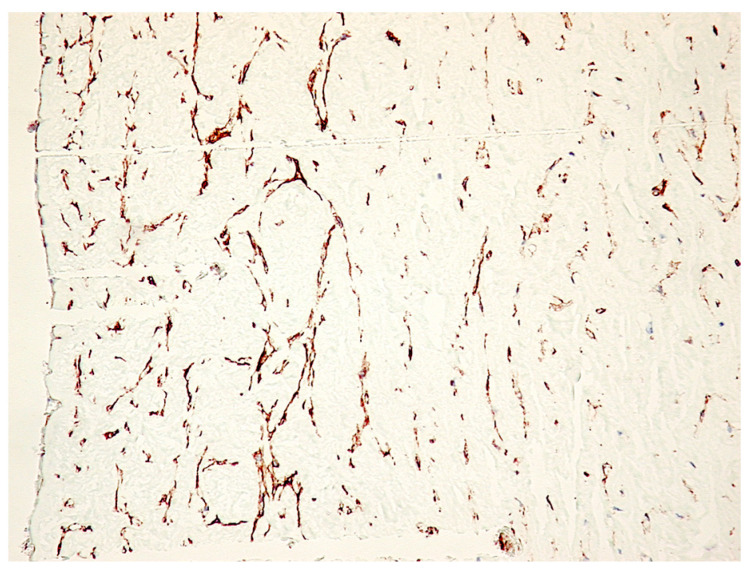
Immunohistochemistry for PDPN of the full-term human umbilical cord. Note the decrease in PDPN-positive structures from the periphery to the center of the cross-section through the umbilical cord. A mixture of isolated PDPN-positive cells with PDPN-positive branched networks may be detected on immunohistochemistry specimens (IHC, 400× magnification).

Immunohistochemically, at the protein level, PDPN expression (assessed by a positive reaction to the D2-40 antibody) was detected over the entire umbilical cord area with a totally different distribution, density and morphology. On the periphery of the umbilical cord, we detected PDPN+ cells, with several projections, highly interconnected with projections from neighboring similar cells and building true networks. Inside this peripheral area of the umbilical cord, PDPN expression defined two regions: (1) the superficial region where PDPN+ cells are present but do not form networks and (2) the deep region where PDPN+ cells form highly interconnected networks. As we moved away from the periphery of the umbilical cord, the density of PDPN+ cells decreased, but their morphology was also changed through fibrocyte-like cells (Figure 1).

**Figure 2:**
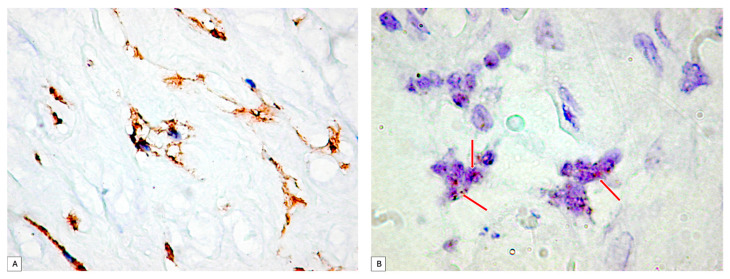
(**A**) PDPN+ cells showing vacuolations of the cytoplasm that merge and outline a tall lumen at the level of the umbilical cord. It should be noted that there is a tendency of PDPN+ cells to branch and make connections with neighboring cells. (**B**) Analysis of PDPN_mRNA presence on umbilical cord specimens. Note the existence of isolated or clustered dotted brown signals (red arrows). PDPN_mRNA expression certified PDPN protein expression assessed by immunohistochemistry.

PDPN+ cells were also distributed inside the wall of the umbilical vessels. From the periphery to the center of the umbilical cord, the branched PDPN+ cells network gradually decreased in number, and they do not form networks, but the PDPN+ cells cytoplasmic vacuolisation still persists in isolated cells from the center of the umbilical cord (Figure 2A).

PDPN_mRNA expression inside the umbilical cord cellular components was assessed first by using the conventional protocol recommended by the manufacturer. Two to four isolated dotted brown positive signals were detected and manually counted according to the manufacturer’s protocol, but the signal intensity was impossible to assess based on this protocol. Related to the number of PDPN_mRNA positive brown dots, they varied in between the umbilical cord peripheral and central areas. PDPN_mRNAscope single signals predominated in isolated cells from the periphery of the umbilical cord, where PDPN_mRNAscope clustered signals were also observed (Figure 2B).

### 3.2. PDPN Protein and PDPN_mRNA Expression in Maternal and Fetal Placenta Structures

The immunohistochemical expression of PDPN was observed in both components of the placenta, namely, the fetal component and the maternal component.

#### 3.2.1. Decidual Cells PDPN Protein Expression

**Figure 3:**
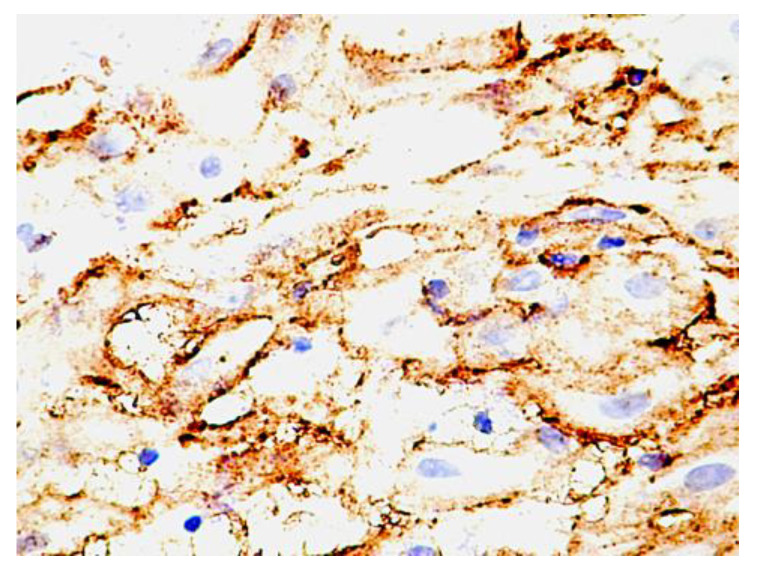
PDPN protein expression in human decidua cells. Note the distribution of the expression, which is maintained as cytoplasmic but is immediately grouped under the membrane. An increased density of isolated positive PDPN cells was also observed in non-decidualized connective tissue.

The decidual cells were intensely positive for PDPN. At this level, the decidual cells showed a positive cell outline for PDPN, suggesting the accumulation of podoplanin at the level of the submembrane peripheral areas in the decidual cells. Cellular structures isolated from the decidual cells were also observed, which presented a branched morphology, like that of the umbilical cord, and kept the tendency to delineate some luminal structures. Compared with PDPN+ cells from the umbilical cord, no vacuolization has been observed in PDPN+ cells from decidua (Figure 3).

#### 3.2.2. Human Fetal Placenta PDPN Protein Expression and Distribution

**Figure 4:**
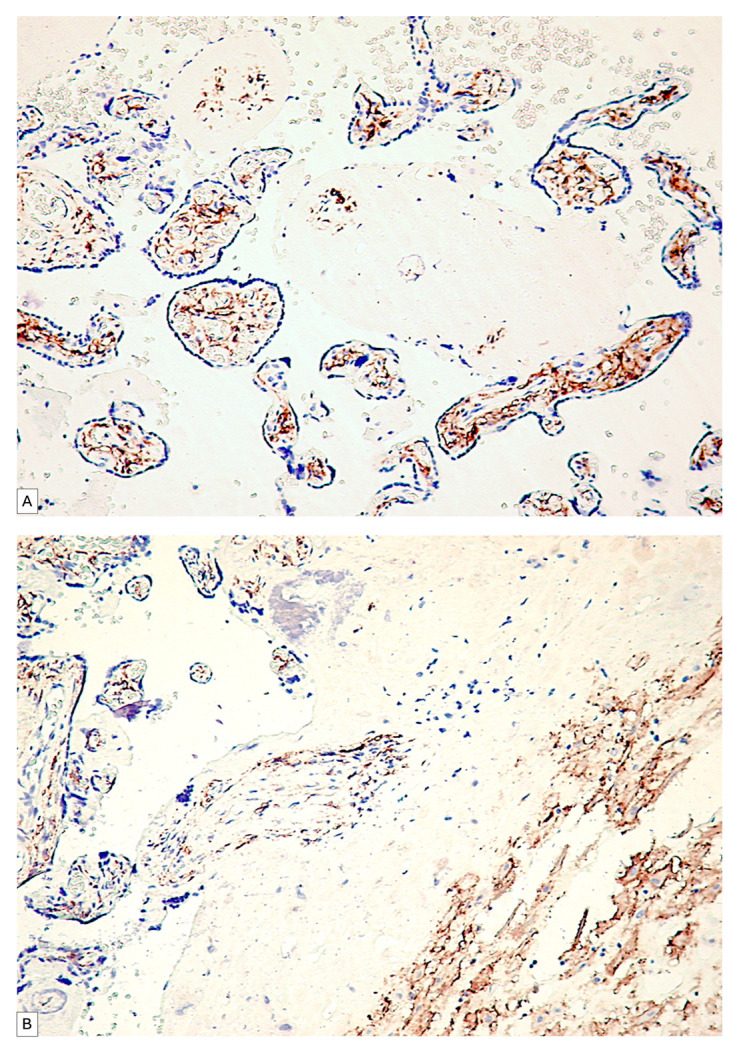
PDPN expression in free placental villi (**A**) and anchoring placental villi (**B**). Note the high density of PDPN-positive cells at the level of free placental villi compared to the anchoring villi, where a decrease in the density of PDPN-positive cells in the center of the placental villi can be observed.

In the human fetal placenta, a strong PDPN protein expression was observed in the placental villi chorion. A higher number of PDPN+ cells was observed inside the free placental villi chorion, while their density progressively decreased for anchoring placental villi chorion. In anchoring placental villi, PDPN expression was limited to the cells at the periphery of the placental villi chorion (Figure 4A,B).

**Figure 5:**
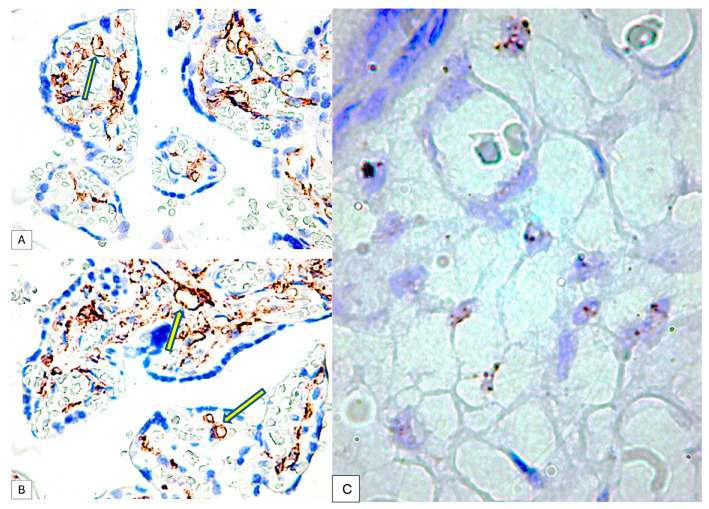
PDPN expression in the connective tissue of tertiary placental villi at term. It should be noted that the blood vessels containing red blood cells are negative for PDPN (**A**), while the mesenchymal cells in the axis of the placental villi are PDPN+ (**B**). Likewise, PDPN-positive immunohistochemical signals with heterogeneous patterns from interconnected networks to circularly arranged PDPN-positive cells close to blood capillaries negative for PDPN were observed in the axis of the placental villi ((**A**,**B**), yellow arrows). mRNA_PDPN detected by the RNAscope in situ hybridization technique. Note the presence of dotted positive brown signals in the cells of the central area, the placental villi chorion. As can be seen, the signals are distinct, with a density that varies between two/cell (which corresponds to a score of +1, according to the established interpretation protocol) and eight positive signals/cell (expression corresponding to a score of +3). However, this interpretation is subjective, which is why we applied digital image analysis to increase the accuracy of the interpretation of the RNAscope signals distribution (**C**).

Blood vessels in the chorion of the placental villi were negative for PDPN, while several PDPN+ cells have heterogeneous patterns arrangements observed predominantly in the tertiary placental villi (Figure 5A,B).

The immunohistochemical expression of PDPN in placental chorion cells was confirmed by RNAscope through the detection of positive signals as brown-stained punctate structures isolated from two to eight per cell until the plus three reaction in which punctate signals were organized in the form of clusters (Figure 5C).

**Figure 6:**
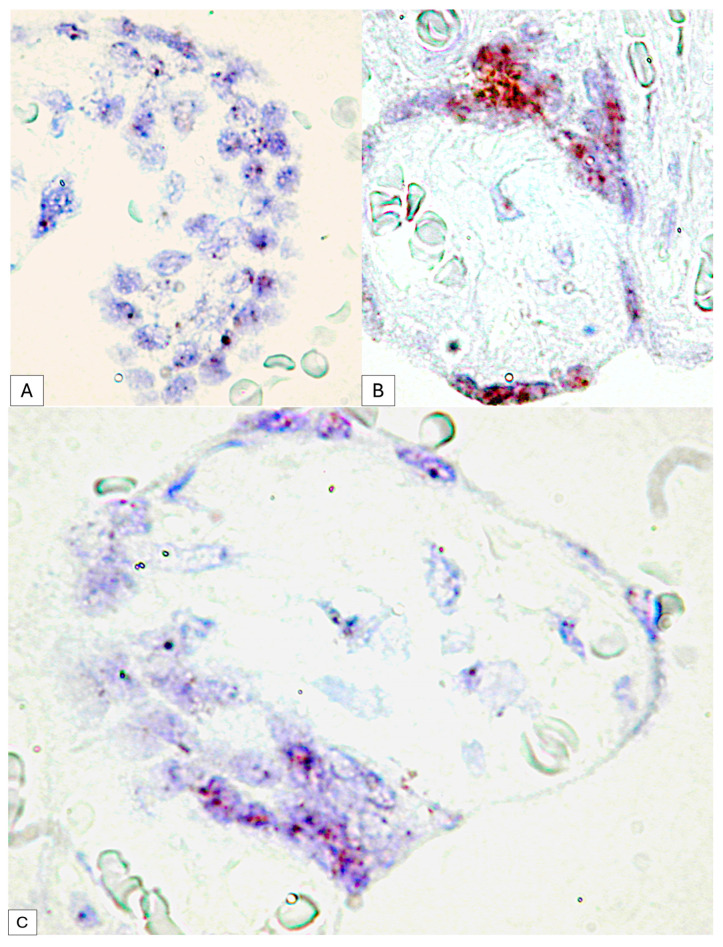
Dotted D2-40 positive signals at the level of syncytiotrophoblast cells (**A**) compared to clusters of RNA scope signals at the level of placental nodes, an aspect that suggests the involvement of PDPN in placental maturation (**B**). Expression variability of mRNA_PDPN on the circumference of the same placental villus. At the agglomeration level of the syncytiotrophoblast cells, the RNAscope signals were agglomerated in the form of clusters, while in the cells arranged on a single layer, the punctate signals were distinct (**C**).

The RNAscope analysis of the trophoblast cells revealed the high density of D2-40 positive dotted signals with different distributions (Figure 6A,B):

• For placental villi syncytiotrophoblast cells, we detected an RNAscope signal highlighted by punctate positive structures between two and four signals per cell nucleus
• Inside placental syncytial knot trophoblastic cells from full-term human placenta, the positive RNAscope signal was much higher than that of the cells of the chorion, and the positive expression of PDPN in the placental knots were observed as condensed punctate clusters.

This variability was observed between the placental villi but also in the trophoblast that delimits the same placental villi, where at the level of the placental knots, the RNAscope signal was organized as clusters, while in the resting trophoblast, two to four dotted distinct signals were observed (Figure 6C).

### 3.3. Digital Image Analysis of Immunohistochemical PDPN Protein Expression and PDPN_mRNA Expression in the Human Umbilical Cord and Placenta

The high density of positive cells, their organization in the form of networks as well as their tendency to form structures with a lumen represent only part of the reasons that led to the use of digital image analysis to increase the accuracy of the interpretation of PDPN expression in the placenta at term and the umbilical cord.

Regarding the immunohistochemical expression of podoplanin in the umbilical cord, the digital image analysis detected a very high variability between the number of positive cells within the umbilical cord; thus, at the periphery of the umbilical cord, we detected a total of 1500 positive cells corresponding to an approximate area of 1857501px until, in the center, we detected a total of 223 cells on a similar microscopic area, about seven times smaller between the periphery of the umbilical cord and mucous connective tissue. Virtually all umbilical cord specimens showed an IHC+ reaction to podoplanin, but the intensity of the expression was different. The quantification of the ALLRED score combining the cell proportion and intensity of expression demonstrated that 88.9% of all evaluated specimens had an Allred score of seven and eight, which supports an intense expression of podoplanin in the umbilical cord and placentas at term.

**Figure 7:**
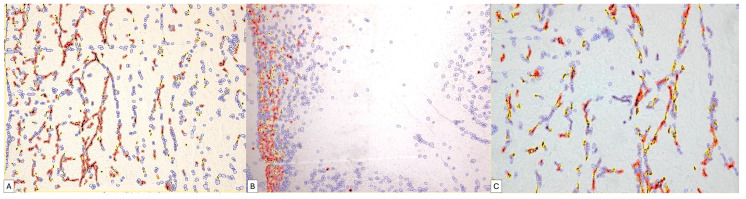
Digital image analysis of umbilical cord specimens immunohistochemically stained with PDPN. Cells with increased intensity are marked with red, those with medium intensity are marked with orange, weakly positive ones are marked with yellow and negative cells are annotated with blue automatically by the image analysis system. Note the increased density of red signals at the periphery of the umbilical cord (**B**). The variability in the expression is dependent on the area of the umbilical cord, progressively decreasing in the middle area of the umbilical cord (**A**) and in its central area (**C**).

Of these cases, 73.3% presented an ALLRED score of seven, the rest being represented by the expression of a maximum ALLRED score of eight (Figure 7).

The positivity percentage of the umbilical cord cells was extremely variable such that from a total number of cells of a specimen, a positive percentage ranging from 3.65% in the center of the umbilical cord to 81.37% at the periphery could be detected in the umbilical cord. A percentage of approximately 61% of the analyzed specimens showed a percentage of positive cells above 80% of the total number of positive cells.

**Figure 8:**
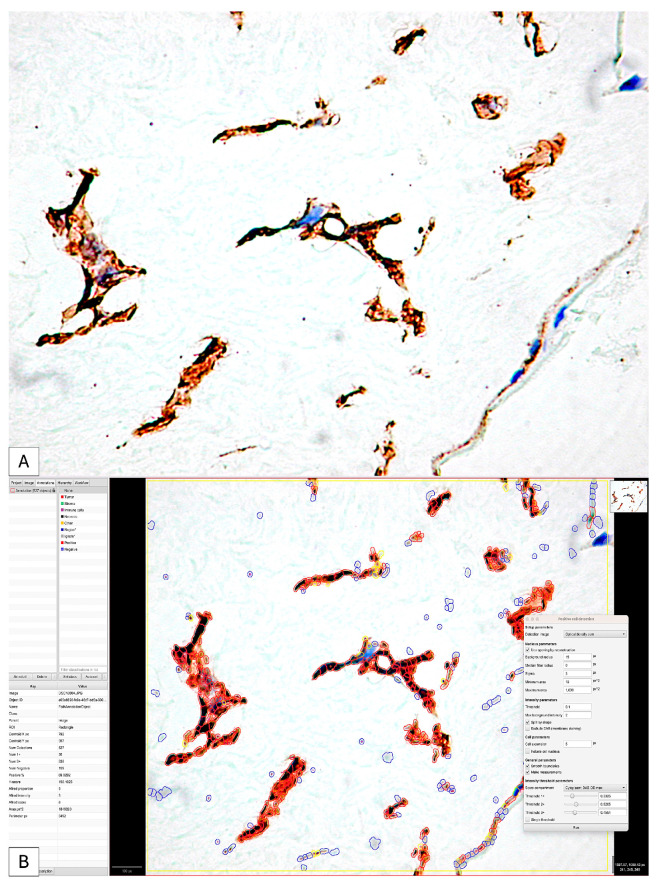
The initial image (**A**) classically interpreted by evaluating the intensity of the immunohistochemical expression of PDPN; it is impossible to evaluate the number, distribution and difference in intensity of positive PDPN cells. The application of digital image analysis allowed not only for the evaluation of the intensity and density of positive PDPN cells but also for the calculation of the H-score and the Allred score by combining the two parameters (**B**) so that the accuracy of the interpretation was significantly higher than that of the classical one.

Regarding the histological score (H-Score), in 83.3% of cases, it had a value above 100 (Figure 8A,B).

Similar results were recorded in the assessment of placental villi, where we also recorded a variability in the Allred score.

**Figure 9:**
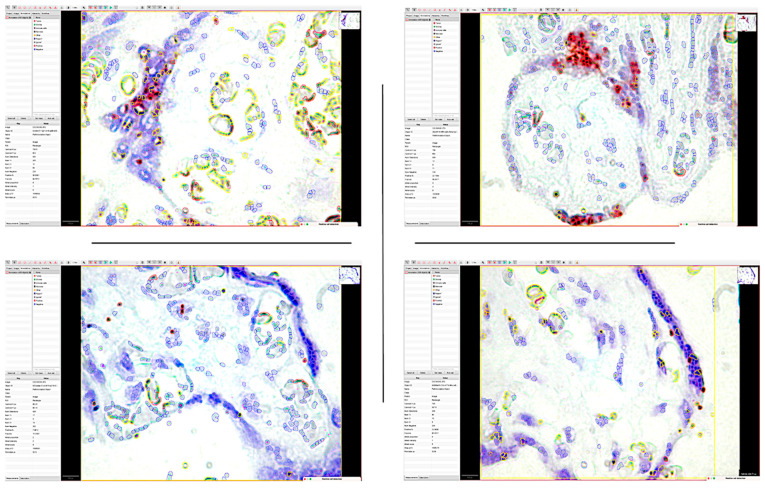
The application of the QuPath digital image analysis system for the quantification of mRNA_PDPN expression reclassified the interpretation of positive dotted signals and is based on the intensity of the gene amplification signal. Thus, the accuracy of identifying medium- and low-intensity signals that DIA could detect has increased. It should be noted that the highest morphometric parameters were detected in the trophoblastic cells of the placental nodes (Allred score of 6), while in the mesenchymal-like cells of the chorionic villus axis, the expression was significantly lower.

We applied the same image analysis system as the RNAscope automated expression quantification method for the first time (Figure 9).

The use of digital image analysis allowed RNAscope to quantify not only the number of positive punctate signals but also the density and intensity of the punctate signal.

For each case, we identified, on the one hand, the number of positive and negative cells in the selected area but also the proportion of positive signals. Inside the same cell, we could separately identify the number of cells that expressed the PDPN gene by categories of intensity.

The percentage of positive cells varied between 7.98% and 79.48% of the total number of detected cells. Of these, the proportion of positive signals recorded an average score in 40% of cases. The intensity of expression was low, being recorded in 73% of cases as one. Overall, the ALLRED score was five or six in 73% of cases.

The statistical analysis of the immunohistochemical expression of PDPN and the gene expression quantified by RNAscope demonstrated a significant correlation between IHC expression and that by RNAscope.

**Figure 10:**
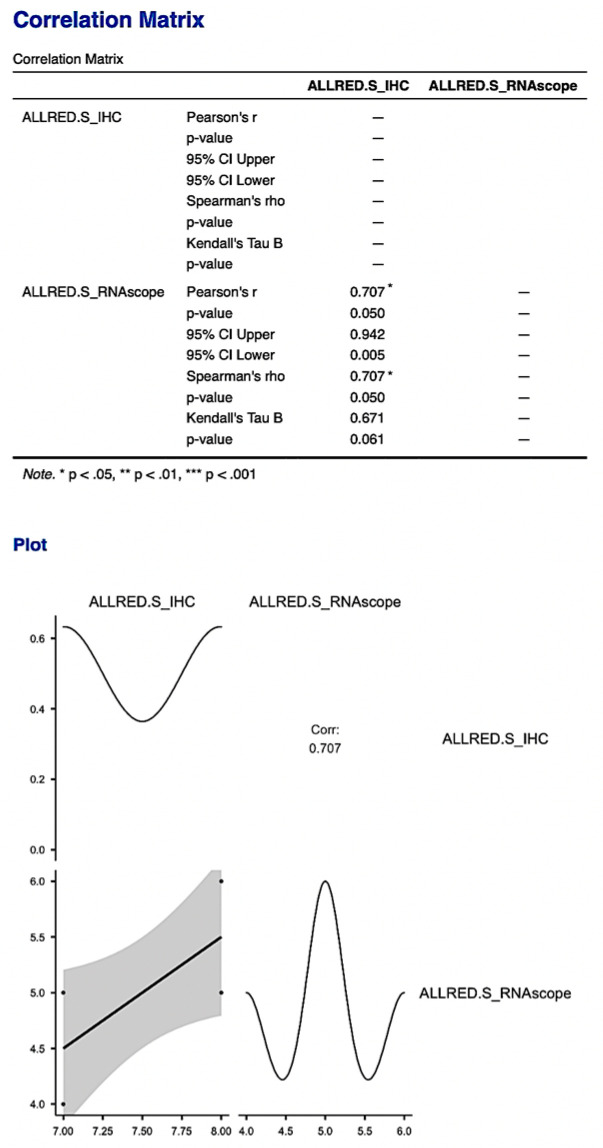
The correlation matrix and correlation diagram between the Allred score obtained from immunohistochemical evaluation and the score obtained from RNAscope evaluation. It is worth noting the statistically significant correlation between the two scores, with the support of the existence and expression of PDPN in the human placenta and umbilical cord.

We compared the Allred score obtained for the immunohistochemical expression of PDPN and the gene expression of mRNA_PDPN and we obtained a statistically significant correlation between IHC and RNAscope, which supports the presence and expression of PDPN in the human placenta and umbilical cord (*p* = 0.05, Figure 10). This result supports and demonstrates the existence and expression of podoplanin in the umbilical cord and placenta at term but also an increased accuracy of PDPN expression evaluation using combined methods.

**Figure 11:**
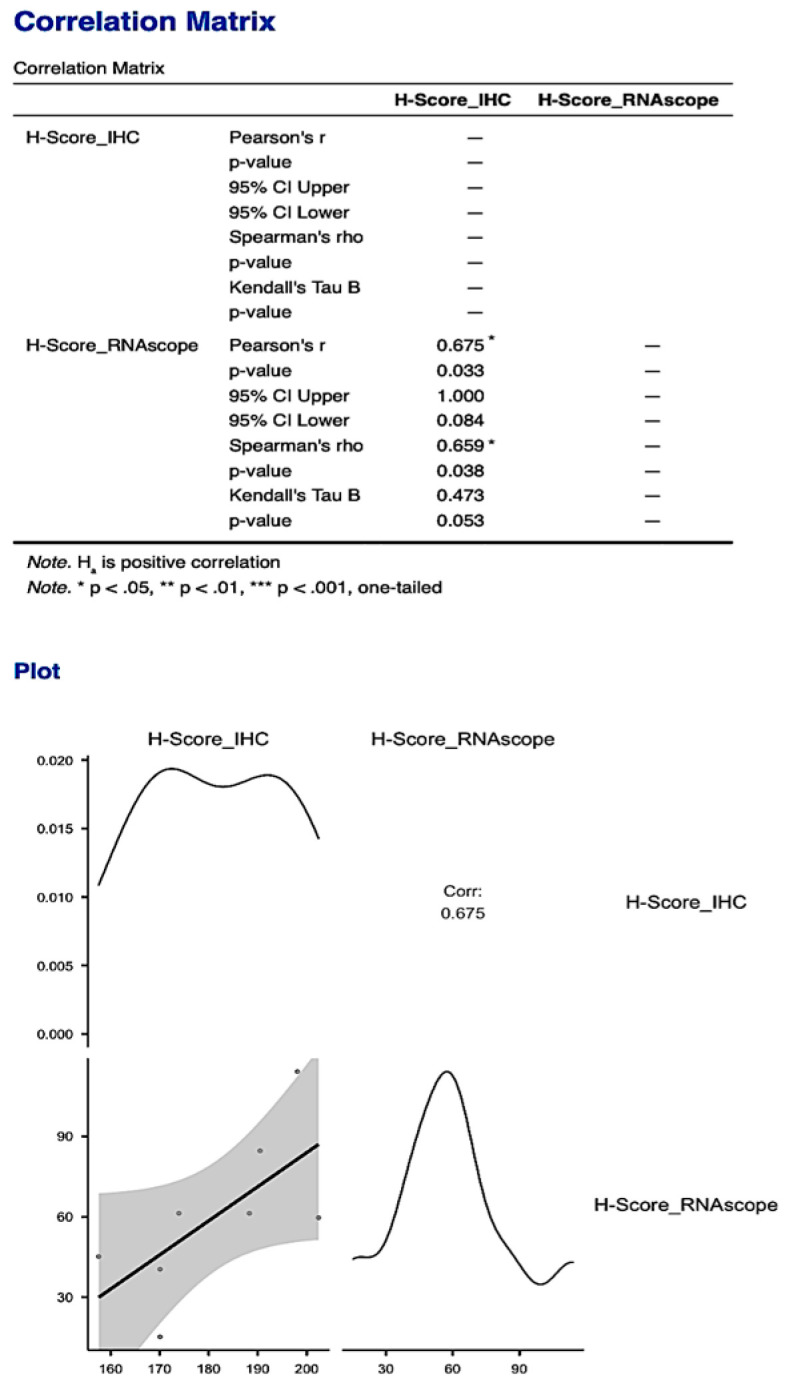
The statistically significant correlation obtained between the histological scores of the two types of analyses for PDPN: protein expression quantified by IHC and gene expression quantified by RNAscope, respectively. The correlation obtained for the H-Score was much stronger compared to the one obtained for the Allred score.

The statistically significant correlation obtained for the Allred score was also supported by obtaining a statistically significant correlation between the histological scores obtained for the two types of analyses performed to quantify PDPN expression (*p* = 0.033, Figure 11).

## 4. Discussion

PDPN expression in the human placenta has been previously reported mainly based on immunohistochemical protein expression, and thus, its expression is still one of the most controversial aspects at the molecular level. In situ hybridization techniques were previously applied on human placenta tissue for the detection of several markers but not for the characterization of PDPN expression in human full-term normal placenta and umbilical cords. Among in situ hybridization techniques, the use of RNAscope on human placenta specimens is very limited, being recently applied for the detection of SARS-CoV-2 RNA [37] or the cell-specific localization of Plasmodium falciparum [38] but not for the detection of the cellular heterogeneity expression of PDPN. The present work is the first report regarding PDPN_mRNA expression and specific distribution in the human normal full-term placenta specimens using the RNAscope technique.

Using RNAscope^®^ hybridization, we were able to visualize cell-specific PDPN- mRNA expression patterns in the formalin-fixed paraffin-embedded (FFPE) specimens. This molecular technique allowed us to report, for the first time, PDPN_mRNA overexpression inside placental knot trophoblastic cells. Further study will be needed for the elucidation of PDPN function in the development of placental knots. At this time, we may speculate that PDPN may help in the placental maturation by the analogy of the placental syncytial knots, similar to the certified function in the human full-term placenta. One mechanism of placental syncytial knots development involves apoptosis. PDPN upregulation may induce neuronal apoptosis in the neuroinflammatory process [39], and a similar mechanism may be involved during syncytial knots development. By applying the RNAscope method, we sharply described the differences in PDPN expression in between the fibroblast-like cells from the placental villi chorion and those of the syncytiotrophoblast.

Our study, partly overlapping with previously published data regarding podoplanin expression in both placental villi and umbilical cords, analyzed the expression of podoplanin in the fetal placenta, maternal placenta and umbilical cord of full-term patients, with no apparent associated pathology. An original aspect of the present study is represented by the PDPN signaling in the umbilical cord cells with a fibroblast-like appearance and the ability to organize in cords.

Lymphangiogenesis was reported by Ortega et al. [40] in patients with chronic venous disease of the lower extremities, which may be associated with structural changes in placental villi. They analyzed the gene expression of PDPN by RT-PCR techniques and reported that it was increased inthe placental villi of women with chronic venous disease of the lower limbs [40].

If, for the placenta, the expression of PDPN has been reported [21,22], the expression of PDPN in the umbilical cord has not yet been demonstrated. Our combined method of IHC, RNAscope and digital image analysis allowed for an accurate description and interpretation of PDPN expression in the umbilical cord mucous connective tissue.

PDPN+ cells are involved in conditions of ischemic hypoxia during pregnancy associated with preeclamptic episodes. Most of the studies carried out to identify lymphatic markers in the placenta were performed using immunohistochemistry methods, whereas other, more sensitive methods, such as in situ hybridization or gene expression techniques, have been rarely used. Regarding the decidua, PDPN expression and the presence of PDPN+ lymphatic vessels have been described outside the decidualization. If the expression of LYVE1 was noted in the early developmental stages of the placenta, the expression of PDPN was noted in the decidual cells [8].

As a paradox, several previous studies of PDPN expression in the human placenta were reported to be performed, mostly associated with pathologic conditions such as preeclampsia [26] or chronic venous disease [40], but its expression in normal human full-term placenta was not described precisely. Here, by combining IHC, RNAscope and digital image analysis, we proved the expression heterogeneity of PDPN in normal full-term human placenta, with its predominant and strong expression inside syncytial placental knots and, to a lesser extent, in the placental villi connective tissue core. PDPN+ fibroblast-like cells have a currently unknown function in the human placenta.

An open-source digital image analyzer known for its customizable features, cross-platform flexibility and easy-to-use interface is called Qu Path. It has been used in a variety of contexts and has been documented in at least 624 papers since its initial release in 2016. Reports of its application in placental tissue are currently scarce, nevertheless. Currently, there are only three papers reporting the use of Qu Path digital image analysis for the assessment of various markers in the human placenta [41,42,43], all of them being published in the last 3 years. None of these papers reported PDPN evaluation in the normal human full-term placenta or umbilical cord. One of these studies [43] referred to the importance of Qu Path analysis in the evaluation of the blood vascular network and syncytial nuclear counting as important parameters of placental villi maturation. While the previous study was based on the use of Qu Path for counting syncytial nuclear aggregates on hematoxylin and eosin stains, our study analyzed PDPN expression in the nuclei of syncytial placental knots. Unfortunately for us, the lack of other similar studies limited the possibility to compare our results with similar ones from other research groups. This may be considered one limitation of the present study, but we consider it important to report our results as a starting point for future similar studies.

Both the PDPN protein expression and PDPN_mRNA expression in the human normal full-term placenta included in the present study were evaluated by generating an ALLRED score and H-Score with Qu Path analysis software. The Allred score and H scoring are two popular visual scoring methods [44]. Tissues are given a % score for staining intensity categories, including none, low, medium and high, using these two procedures. Ordinal (semiquantitative) data are produced as a result. Because digital image analysis makes it possible to provide quantitative data rather than ordinal data, robust results with more accurate, linear correlations with biological and clinical outcomes are produced. Previous studies reported PDPN placental expression by using visual, manual evaluation with a high grade of subjectivity based on non-standardized interpretation protocols [22]. Here, we provide the first automatically generated ALLRED and H-Scores values for PDPN expression in the normal full-term human placenta. Due to the high accuracy of the assessment based on these scores in the normal placental tissue, these values may be considered and used as reference values for comparing PDPN expression from normal placental tissue with values obtained for placental pathologic conditions in future studies for the better characterization of PDPN’s role in such pathologies.

## 5. Conclusions

PDPN is expressed in the human placenta at term and the umbilical cord both at the protein level and the mRNA level. PDPN+ structures have a different distribution and organization in the fetal, maternal placenta and umbilical cord at term. The combined method of assessing PDPN expression by immunohistochemistry and RNAscope increases the accuracy of assessing the PDPN of the placenta and reduces the existing controversies regarding the presence of PDPN in the human placenta and umbilical cord.

Frequently, PDPN+ cells had a tendency for the intracytoplasmic vacuolization and fusion of these vacuoles, which led to the outline of structures with a distinct lumen. The PDPN+ luminal structures did not contain red blood cells but have a patent lumen lined by PDPN-positive cells.

The decidual cells intensely expressed PDPN at the protein and gene levels. At the protein level, the PDPN distribution had a particular character, being distributed in a strictly submembrane manner in all decidual cells.

The highest Allred scores and H Scores were evaluated in the placental syncytial knots from the full-term placenta. This aspect has not been described before and suggests PDPN involvement in the maturation of the placental villi.

## Data Availability

The data presented in this study are available on request from the corresponding author. The data are not publicly available due to protection data laws from our area and from our university.
